# Anatomical liver segmentectomy 2 for combined hepatocellular carcinoma and cholangiocarcinoma with tumor thrombus in segment 2 portal branch

**DOI:** 10.1186/1477-7819-10-22

**Published:** 2012-01-25

**Authors:** Hiromichi Ishii, Takuma Kobayashi, Michihiro Kudou, Masumi Nishimura, Atsushi Toma, Kenji Nakamura, Takeshi Mazaki, Tsuyoshi Itoh

**Affiliations:** 1 Division of Surgery, Kyoto Prefectural Yosanoumi Hospital, 481 Otokoyama, Yosano-cho, Yosa-gun, Kyoto 629-2261, Japan; 2 Division of Pathology, Kyoto Prefectural Yosanoumi Hospital, 481 Otokoyama, Yosano-cho, Yosa-gun, Kyoto 629-2261, Japan

**Keywords:** anatomical segmentectomy 2, portal vein tumor thrombus, combined hepatocellular carcinoma and cholangiocarcinoma, modified selective hepatic vascular exclusion

## Abstract

**Background:**

Hepatic resection is the only effective treatment for combined hepatocellular carcinoma and cholangiocarcinoma.

**Case presentation:**

A 52-year-old man was preoperatively diagnosed with hepatocellular carcinoma in segment 2 with tumor thrombus in the segment 2 portal branch. Anatomical liver segmentectomy 2, including separation of the hepatic arteries, portal veins, and bile duct, enabled us to remove the tumor and portal thrombus completely. Modified selective hepatic vascular exclusion, which combines extrahepatic control of the left and middle hepatic veins with occlusion of left hemihepatic inflow, was used to reduce blood loss. A pathological examination revealed combined hepatocellular carcinoma and cholangiocarcinoma with tumor thrombus in the segment 2 portal branch. No postoperative liver failure occurred, and remnant liver function was adequate.

**Conclusion:**

The separation method of the hepatic arteries, portal veins, and bile duct is safe and feasible for a liver cancer patient with portal vein tumor thrombus. Modified selective hepatic vascular exclusion was useful to control bleeding during liver transection. Anatomical liver segmentectomy 2 using these procedures should be considered for a patient with a liver tumor located at segment 2 arising from a damaged liver.

## Background

Combined hepatocellular carcinoma and cholangiocarcinoma (cHCC-CC) is an uncommon primary liver cancer subtype [1] and is difficult to correctly diagnose preoperatively. Most patients with cHCC-CC are preoperatively misdiagnosed with hepatocellular carcinoma (HCC) or cholangiocarcinoma (CC) including our present patient. Hepatic resection leads to improved survival in patients with cHCC-CC [2-5] or HCC with portal vein tumor thrombus (PVTT) [6,7]. In patients with liver cirrhosis, extended liver resection for liver cancer is sometimes not feasible because of decreased liver functional reserve; therefore, anatomical segmentectomy or limited non-anatomical hepatectomy must be performed. We herein report an anatomical liver segmentectomy 2 surgical procedure successfully performed for a patient with cHCC-CC and PVTT in the segment 2 portal branch (P2) root arising from liver cirrhosis due to hepatitis B virus. The Brisbane 2000 terminology of liver anatomy and resections was used.

## Patient and Method

**Figure 1:**
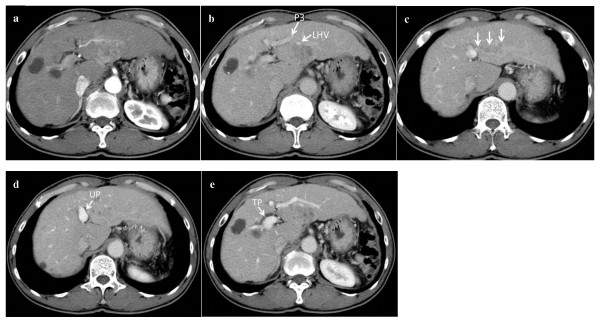
**Dynamic computed tomography revealed a tumor in segment 2 of the liver, which was high and low density in the arterial (a) and venous phases (b), respectively, and portal vein tumor thrombus in the portal branch root of segment 2 (arrow) (c)**. Portal vein tumor thrombus did not invade to umbilical and transverse portions (UP and TP) of the left portal vein (d, e). P3, portal branch of segment 3; LHV, left hepatic vein.

A 52-year-old man was admitted to our hospital for treatment of liver tumor. Five years previously, he underwent splenectomy for hypersplenism due to liver cirrhosis, partial gastrectomy for early gastric cancer, and cholecystectomy for a gallstone. Abdominal dynamic computed tomography (CT) revealed a high- and low-density lesion in the arterial and venous phases, respectively (Figure 1a and 1b). Although this tumor was fed slightly by the left hepatic artery of segment 3, the tumor location was liver segment 2 only. The lesion was 4 cm in diameter and apposed the left hepatic vein. CT also revealed PVTT in the root of P2 (Figure 1c), excluding the umbilical and transverse portion of the left portal vein (Figure 1d and 1e). Based on the CT volumetric study excluding the volume of the tumor, the volumes of total liver, left hemiliver, left lateral section, and segment 2 were 780, 351 (45% of the total liver), 320 (41%), and 164 cm^3 ^(21%), respectively. The Child-Pugh classification status and the degree of liver damage scoring system designed by the Liver Cancer Study Group of Japan [8] were determined as class A (5 points) and class A, respectively, based on laboratory data obtained at hospitalization (total bilirubin: 1.2 mg/dL, albumin: 4.5 g/dL, prothrombin activity: 98.4%, aspartate aminotransferase: 31 IU/L, alanine aminotransferase: 27 IU/L, and indocyanine green retention value at 15 min after intravenous injection (ICG-R15): 24%). Considering the impaired liver function indicated by an ICG-R15 of 24% and the hypertrophied left lateral section, we decided to perform anatomical liver segmentectomy 2 rather than left hepatectomy or left lateral sectionectomy.

### Surgical technique

**Figure 2:**
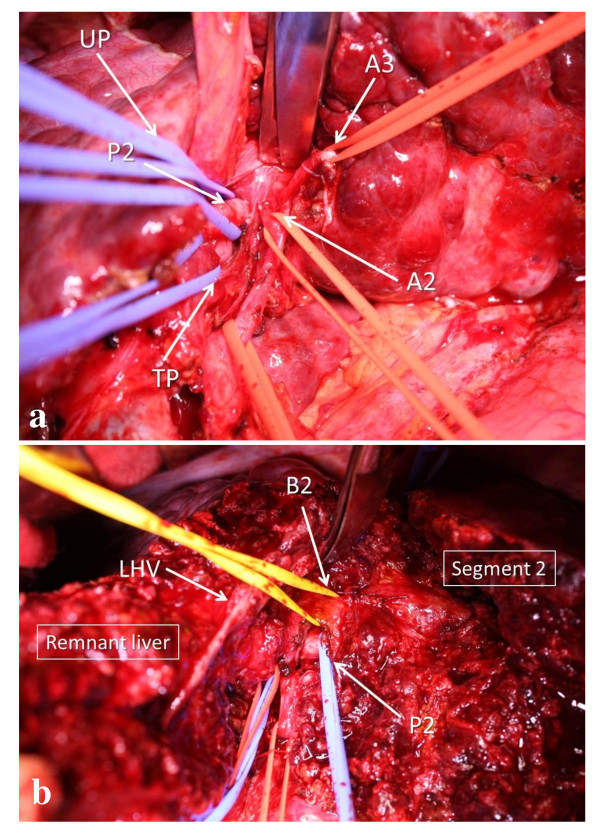
**Left hepatic arteries of segment 2 (A2) and 3 (A3), umbilical and transverse portions (UP and TP) of the left portal vein, and portal branch of segment 2 (P2) were encircled (a)**. Segment 2 and remnant liver were connected only through the portal branch and bile duct of segment 2 (P2 and B2) (b). LHA, left hepatic artery. LHV, left hepatic vein.

An upper median skin incision was made, and the round ligament was ligated and divided. We routinely conduct intraoperative ultrasonography for hepatectomy to define the tumor location and vessels to be manipulated for resection. The left lateral section was mobilized by incising the falciform, left coronary, and left triangular ligaments. The left lateral section was retracted to the right, and the Arantius canal was transected on both sides of the umbilical portion of the left portal vein and left hepatic vein. Arantius canal transaction on the side of the left hepatic vein with retraction of the mobilized left lateral section made it easier to encircle the extrahepatic portion of the common trunk of the middle and left hepatic veins. The common bile duct, left portal vein, and left hepatic artery feeding segments 2 and 3 were encircled in the hepatic hilum separately. The round ligament was retracted anteriorly, and the umbilical portion of the left portal vein was exposed by dissecting the serosa of the umbilical fissure. The left hepatic arteries of segment 2 (A2) and 3 (A3) were encircled at the left side of the umbilical portion of the left portal vein after thorough mobilization, and encircling of the umbilical and transverse portions of the left portal vein and P2 by sacrificing one of the portal branches supplying segment 1 (Figure 2a). After A2 was ligated and divided, the liver parenchyma was dissected using the Cavitron Ultrasonic Surgical Aspirator (CUSA^®^) under left hemihepatic vascular occlusion [9] from the periphery toward the vena cava along the demarcation line separating segments 2 and 3 that appeared by clamping the umbilical portion of the left portal vein and A3. Bleeding from the left hepatic vein during transection was controlled using an extrahepatic clamp on the common trunk of the middle and left hepatic veins. After liver transection, segment 2 and the remnant liver were connected through only P2 and the segment 2 bile duct (B2) (Figure 2b). Following ligation and division of the B2, P2 was incised at its origin after clamping the umbilical and transverse portions of the left portal vein to ensure complete removal of the tumor thrombus under direct vision. The P2 stump was then closed with continuous sutures. The cut surface of the liver with exposure of the left hepatic vein, indicating the anatomical landmark dividing segments 2 and 3.

Operative and left hemihepatic vascular occlusion times were 419 and 92 min, and blood loss during the operation and liver dissection were 939 and 500 ml. No blood transfusions were required during or after operation.

The postoperative course was good, with peak levels of serum aspartate aminotransferase, alanine aminotransferase, and total bilirubin of 264 IU/L, 243 IU/L, and 1.6 mg/dl. A left portal vein thrombus was revealed by CT and ultrasonography on postoperative day 8; however, this thrombus disappeared with anti-coagulant therapy. He was discharged 17 days after surgery.

**Figure 3:**
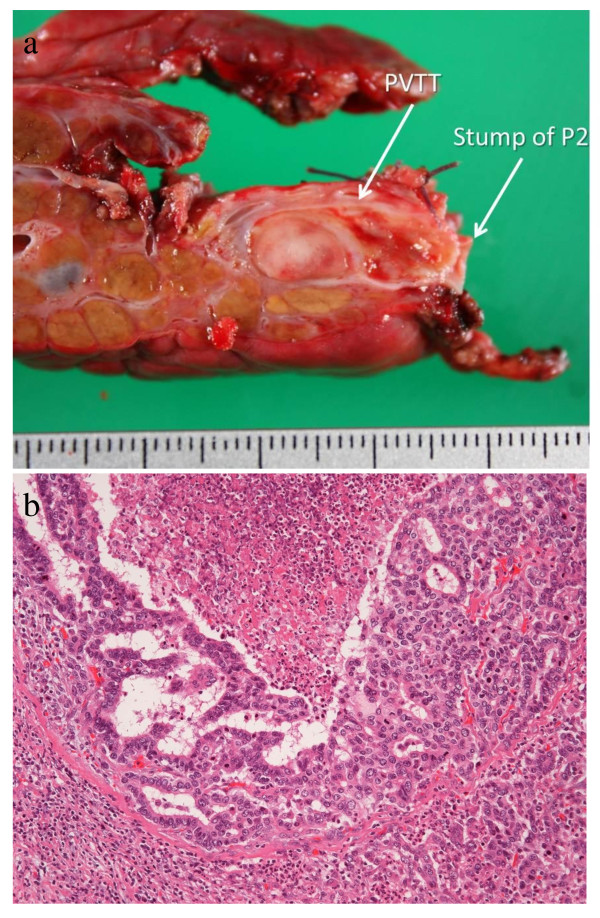
**Portal vein tumor thrombus (PVTT) in the segment 2 portal branch (P2) (a)**. Microscopically, the tumor was combined hepatocellular carcinoma and cholangiocarcinoma (Hematoxylin and eosin staining; magnification ×10) (b).

Macroscopically, PVTT was found in P2, and the surgical margin of PVTT was 1 mm (Figure 3a). Microscopically, the tumor was composed of HCC with trabecular structure and adenocarcinoma (Figure 3b), suggesting cHCC-CC. The patient is alive 9 months after surgery, and has been undergoing systemic chemotherapy using orally administered S-1 for multiple lymph nodes metastasis.

## Discussion

The World Health Organization [10] defined cHCC-CC as a rare tumor containing unequivocal elements of intimately admixed HCC and CC. This tumor is distinguished from separate HCC and CC arising within the same liver. Patients with cHCC-CC and HCC are clinicopathologically similar in average age at the time of diagnosis (i.e., 50-60 years old), with a male predominance, viral hepatitis, elevated α-fetoprotein, and liver cirrhosis [3,4,11], and clinicopathological features in our case were similar to the above data. It is difficult to make a differential diagnosis of cHCC-CC or HCC; however, therapeutic options for patients with these tumors are almost the same, and liver resection leads to improved survival for both tumors [2-7].

Because a liver tumor located in segment 2 with PVTT in P2 is thought to readily metastasize through the portal venous flow to segments 3 and 4, left hepatectomy is the first choice of treatment. Left lateral sectionectomy is the second choice because the tumor was close to the left hepatic vein. However, in patients with liver cirrhosis, the left lateral section hypertrophies, and left hepatectomy or left lateral sectionectomy are sometimes not feasible because of decreased liver functional reserve and the relatively large volume of the left hemiliver or left lateral section [12]. In our case, the left hemiliver and left lateral section became large (45% and 41% of the total liver, respectively) and the ICG-R15 level was relatively high (24%). And we judged preoperatively that we can exfoliate the tumor from the left hepatic vein. Therefore, anatomical liver segmentectomy 2 rather than left hepatectomy or left lateral sectionectomy was selected because it optimizes the balance between oncological requirements and the need to spare functioning liver parenchyma. Although preoperative surgical planning was evaluated by two-dimensional CT in our case, we think that three-dimensional CT computer-assisted preoperative surgical planning may be helpful for anatomical liver segmentectomy 2 [13]. In anatomical segmentectomy 2, transection of the glissonean pedicle that feeds segment 2 [12] and intraoperative ultrasound-guided blunt compression of the segment 2 portal branch [14] have been reported; however, these techniques are not suitable in liver cancer patients with PVTT in the root of P2, including our patient. In such cases, complete removal of PVTT is extremely important to prevent early tumor recurrence; therefore, after A2, A3, P2, and umbilical and transverse portions of the left portal vein were separated, the liver was dissected, and PVTT was removed under direct vision. It is easy to ligate and divide the origin of P2 before liver transection; however, we think that it is safer and more certain to incise the origin of P2 and suture the stump of P2 after liver transection than before liver transection. This separation method could be adapted for segmentectomy 3 and segmentectomy 3 and 4 [15]. Although several reports [16,17] have demonstrated the feasibility and safety of laparoscopic anatomic resection based on three-dimensional CT images recently, we performed anatomical liver segmentectomy 2 by open surgery because laparoscopic approach to hepatic tumors remains a challenge, and the patient in present report underwent upper abdominal surgeries (splenectomy, partial gastrectomy, and cholecystectomy). Left hemihepatic vascular occlusion, limited to 30 min followed by 5 min of perfusion, is useful to prevent blood loss originating from hepatic inflow [9]; however, it is difficult to control retrograde bleeding from the left hepatic vein using this maneuver. Selective hepatic vascular exclusion (SHVE), which combines inflow vascular occlusion (Pringle maneuver) with extrahepatic control of the major hepatic veins, overcomes the drawbacks of backflow bleeding of the Pringle maneuver [18]. In segmentectomy 2 or 3, modified selective hepatic vascular exclusion (m-SHVE), which combines extrahepatic control of the middle and left hepatic veins with left hemihepatic inflow occlusion, is sufficient to reduce both backflow and inflow bleeding. In our case, this m-SHVE procedure contributed to reduce bleeding during liver dissection and was not associated with hemodynamic changes. SHVE has been used in major liver resections to control intraoperative bleeding [19,20]; however, this technique has not been reported and studied in anatomical liver segmentectomy 2, to our knowledge. Furthermore, although it is reported in previous publications that SHVE entails Pringle maneuver and extrahepaic clamping of major hepatic veins [19,20], m-SHVE which entails not Pringle maneuver but hemihepatic inflow occlusion has not been reported. Therefore, anatomical liver segmentectomy 2 with m-SHVE is the novel technique. We think that this m-SHVE is the effective technique in anatomical liver segmentectomy 2 and can be adapted also for anatomical liver segmentectomy 3. Intraabdominal adhesion resulting from previous surgery was very severe, and then, about 120 min were required and blood loss was about 400 ml during exfoliation of this adhesion. Therefore, we think that the operative time (419 min) and the amount of blood loss (939 ml) in our case seem in the tolerance. The liver transection time was relatively longer because of the hard texture of liver parenchyma resulting from liver cirrhosis. Because postoperative liver function was adequate in our case, anatomical segmentectomy 2 is feasible to preserve remnant liver function in selected patients with liver cirrhosis.

Survival of patients with cHCC-CC was significantly poorer than that of HCC or CC patients [3-5], and the PVTT was found to be significant predictor of poor outcome [3,5]. In our case, the patient is alive 9 months after surgery with lymph nodes metastasis. Although limited hepatectomy was performed, the patient has no recurrence in the remnant liver. Therefore, in selected patients with liver cirrhosis, limited hepetectomy including anatomical segmentectomy 2 may be an appropriate operation even if patients have the PVTT.

## Conclusions

The hepatic arteries, portal veins, and bile duct separation method is a useful approach for a liver cancer patient with PVTT, and m-SHVE is effective for minimizing bleeding during liver resection of segmentectomy 2. Anatomical liver segmentectomy 2 contributes to preserve remnant liver function; however, further investigation is needed to evaluate the overall survival rates in patients who undergo segmentectomy 2.

## Consent

Written informed consent was obtained from the patient for publication of his clinical details and accompanying images. A copy of the written consent is available for review by the Editor of this journal.

## Competing interests

The authors declare that they have no competing interests.

## Authors' contributions

HI wrote the first draft of this report. HI, MN, and TI performed the operation. TM performed the pathological examination. HI is the guarantor of the paper. All authors read and approved the final manuscript.
